# Antimicrobial resistance in bloodstream isolates of *Escherichia coli* and *Staphylococcus aureus* from a provincial hospital, Cambodia, 2020–2022

**DOI:** 10.5365/wpsar.2025.16.4.1182

**Published:** 2025-11-24

**Authors:** Sivhour Chiek, Vichet Orn, Rina Dork, Sreypeou Hem, Sophanna Phai, Phally Kheng, Bunranai Thoeun, Seila Kak, Sidonn Krang, Sovann Ly, Sopheap Oeng, Paul Turner

**Affiliations:** a Battambang Provincial Referral Hospital, Battambang, Cambodia.; b Communicable Disease Control Department, Ministry of Health, Phnom Penh, Cambodia.; c Management4health and Integrated Quality Laboratory Services, Phnom Penh, Cambodia.; d Cambodia–Oxford Medical Research Unit, Angkor Hospital for Children, Siem Reap, Cambodia.; e Centre for Tropical Medicine and Global Health, Nuffield Department of Medicine, University of Oxford, Oxford, United Kingdom of Great Britain and Northern Ireland.

## Abstract

Antimicrobial resistance (AMR) is a global concern. However, in Cambodia, as in other countries in the World Health Organization’s Western Pacific Region, the magnitude of the problem is largely unknown. Thus, this study aimed to determine the prevalence of AMR in common pathogens, namely *Escherichia coli* and *Staphylococcus aureus,* isolated from blood cultures at one provincial hospital, a national sentinel site for AMR surveillance, during a 3-year period. Sample processing and analysis were conducted at the hospital’s on-site microbiology laboratory. Blood cultures were processed manually, and conventional methods were used for bacterial identification. Antibiotic susceptibility testing (AST) was performed by disk diffusion and Etest minimum inhibitory concentration measurement, in accordance with current Clinical and Laboratory Standards Institute guidelines. Blood culture data from 1 January 2020 to 31 December 2022 were extracted from the hospital’s microbiology database and, for the AST analysis, deduplicated to include results only for the first isolate per patient per year. Of 6102 blood cultures collected, 529 (9%) were positive. The most common blood culture pathogens found were *E. coli* (150, 28% of positive isolates) and *S. aureus* (65, 12% of positive isolates). For *E. coli*, resistance to ceftriaxone was detected in 110/148 (74%) isolates and resistance to imipenem in 3/147 (2%). For *S. aureus*, 18/56 (32%) isolates were methicillin-resistant, but vancomycin resistance was not detected. These rates of resistance to first-line treatments are of concern and have the potential to negatively impact patient outcomes.

Antimicrobial resistance (AMR) has emerged as a significant global public health threat, with nearly 5 million deaths linked to resistant bacteria in 2019. (*1*) Bacterial AMR also leads to treatment difficulties and longer hospital stays, resulting in increased health-care costs. (*2*)

It is essential to understand the true scale of AMR to inform risk management and identify opportunities for timely mitigation. The World Health Organization (WHO) established the Global Antimicrobial Resistance and Use Surveillance System (GLASS) in 2015 to enable countries to collect and share microbiological and antimicrobial use data. (*3*) This surveillance system targets six bacterial species commonly isolated from clinical samples globally, including *Escherichia coli* and *Staphylococcus aureus*, both dominant causes of bloodstream infections.

By the end of 2022, 92 countries had contributed AMR data to GLASS, including 10 from WHO’s Western Pacific Region. Despite the growth of the GLASS database, large gaps remain in the global AMR data set. (*1*) Although Cambodia has been reporting some data to GLASS since 2018, according to a review published in 2019, the scale of the AMR problem in Cambodia remains largely unknown. (*4*) This study aimed to provide contemporary data about AMR in Cambodia by determining its prevalence in *E. coli* and *S. aureus* isolated from blood cultures submitted between 2020 and 2022 to Battambang Provincial Referral Hospital (BPRH), one of several sentinel sites belonging to Cambodia’s national AMR surveillance system.

## Methods

### Study site

BPRH, located in the north-west of the country, is a complete level-3 health-care facility, with a catchment area of around 1 million people. The hospital has 390 beds and departments for adult medicine, surgery, paediatrics, intensive care and obstetrics. The on-site microbiology laboratory processes around 3600 clinical samples per year from hospitalized patients and those attending surrounding health-care facilities. The laboratory participates in the national external quality assurance programme.

### Blood culture practices and processing

National standard operating procedures for AMR surveillance recommend blood culture for hospitalized patients with fever and a suspected bacterial infection. At BPRH, blood cultures are processed manually. For adults, 10 mL of blood are collected for culture from two different sites and inoculated into a pair of 100-mL aerobic culture bottles (containing brain–heart infusion broth + 0.025% sodium polyanethol sulfonate). For children, 1–5 mL of blood are inoculated into a single 50-mL aerobic bottle. The blood culture bottles are then incubated in a static incubator at 35 °C (± 2 °C) for up to 7 days. All bottles are checked daily for signs of growth, including for turbidity, gas bubbles and haemolysis. If growth is detected, the bottle is Gram stained and subcultured onto a range of media. Additionally, blind subculture to chocolate agar and Gram stain are performed after 1 day of incubation for all bottles. Bacterial identification is done using conventional methods: catalase and coagulase for *S. aureus*; and oxidase, indole, and a panel of five biochemical tests for *E. coli* and other Gram-negative bacteria.

Antimicrobial susceptibility tests (ASTs) are done by disk diffusion and measurement of Etest minimum inhibitory concentration, following Clinical and Laboratory Standards Institute guidelines and standards (M02 and M100). (*5*, *6*) Specific species or groups of species were tested against standard panels of antimicrobial agents. Susceptibility to cefoxitin was tested as a surrogate agent for oxacillin and methicillin to report *S. aureus* resistance results for cloxacillin and cefazolin. Vancomycin was tested against *S. aureus* isolates only when cefoxitin resistance was detected.

### Data analysis

Microbiology data were extracted from the national laboratory information system into a Microsoft Excel spreadsheet. Data were deduplicated to include only those results obtained from the first isolate per patient per year for each species (*E. coli* and *S. aureus*). Data summaries and graphs were generated using R, version 4.3.0 (R Core Team, Vienna, Austria), with the AMR, Harrell Miscellaneous (known as Hmisc) and tidyverse packages. (*7*) The χ^2^ test was used to explore trends in resistance to key GLASS surveillance antimicrobials (i.e. ceftriaxone for *E. coli* and methicillin for *S. aureus*).

## Results

### Hospital and laboratory summary

Between 1 January 2020 and 31 December 2022, 52 326 patients were admitted to BPRH: 17 947 in 2020, 16 312 in 2021 and 18 067 in 2022. During this period, 6102 blood cultures were processed by the microbiology laboratory: 5107 (84%) from hospitalized patients and 995 (16%) from patients attending external health-care facilities.

### Blood culture data

Growth was detected in 826/6102 (14%) blood cultures. Of these, 297 were contaminated by skin flora (growth of coagulase-negative staphylococci, *Micrococcus* spp., *Corynebacterium* spp. or *Bacillus* spp.), leaving a total of 529 true positives. *E. coli* and *S. aureus* were the most common pathogens isolated from blood cultures, detected in 150 (28%) and 65 (12%) of positive cultures, respectively (**Table 1**).

**Table 1:** The 10 most common pathogens isolated from blood cultures analysed at a sentinel surveillance site for antimicrobial resistance, Cambodia, 2020–2022 (*n* = 529)

Species or species group	No. of isolates	% of true positive isolates
***Escherichia coli***	**150**	**28.4**
***Staphylococcus aureus***	**65**	**12.3**
**Burkholderia pseudomallei**	**61**	**11.5**
**Klebsiella pneumoniae**	**47**	**8.9**
**Non-fermenting Gram-negative rodsa**	**40**	**7.6**
**Acinetobacter spp.**	**24**	**4.5**
**Other Enterobacteralesb**	**16**	**3.0**
**α-haemolytic (viridans) streptococci**	**12**	**2.3**
**Pseudomonas aeruginosa**	**12**	**2.3**
**Aeromonas spp.**	**9**	**1.7**
**Enterococcus spp.**	**9**	**1.7**

^a^ This group refers to oxidase-positive non-lactose-fermenting Gram-negative bacilli not speciated by available tests.

^b^ This group refers to oxidase-negative Gram-negative bacilli not speciated by available tests (i.e. excluding *E. coli* and *K. pneumoniae*).

### Antimicrobial susceptibility data

#### Escherichia coli

**Fig. 1:**
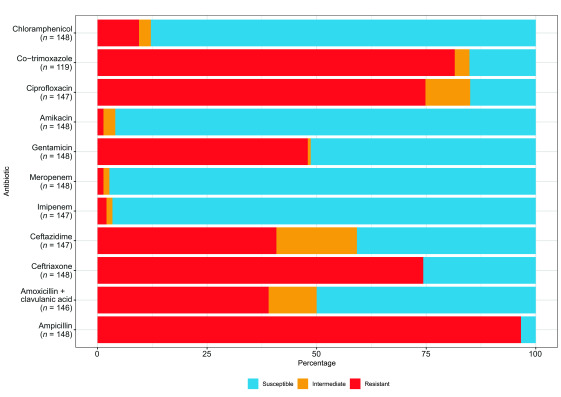
Antimicrobial resistance in *Escherichia coli* blood isolates, Battambang, Cambodia, 2020–2022

After deduplication, there were 148 *E. coli* blood culture isolates with AST data (**Fig. 1**). Resistance to the following was common: ampicillin (143/148, 97%; 95% confidence interval [CI]: 92–99%), ceftriaxone (110/148, 74%; 95% CI: 67–81%), co-trimoxazole (97/119, 82%; 95% CI: 73–88%) and ciprofloxacin (110/147, 75%; 95% CI: 67–82%). Resistance to the following was rare: amikacin (2/148, 1%; 95% CI: 0–5%), imipenem (3/147, 2%; 95% CI: 0–6%) and meropenem (2/148, 1%; 95% CI: 0–5%). AST results stratified by patient’s age (< 18 years, ≥ 18 years) are summarized in **Table 2**. There was no trend in resistance to ceftriaxone over time (73% [30/41] in 2020, 77% [49/64] in 2021, and 72% [31/43] in 2022; χ^2^ for trend *P* = 0.90) (data not shown).

[**insert** Figure 1]

**Table 2:** Antimicrobial resistance in *Escherichia coli* blood isolates, stratified by patient’s age group, Cambodia, 2020–2022

Antimicrobial class	Antibiotic	Adult^a^				Paediatric^a^			
		Total	Susceptible	Intermediate	Resistant	Total	Susceptible	Intermediate	Resistant
**Amphenicols**	**Chloramphenicol**	**143**	**125 (87.4)**	**4 (2.8)**	**14 (9.8)**	**5**	**5 (100.0)**	**0 (0)**	**0 (0)**
**Folate antagonists**	**Co-trimoxazole**	**114**	**16 (14.0)**	**4 (3.5)**	**94 (82.5)**	**5**	**2 (40.0)**	**0 (0)**	**3 (60.0)**
**Fluoroquinolones**	**Ciprofloxacin**	**142**	**20 (14.1)**	**14 (9.9)**	**108 (76.1)**	**5**	**2 (40.0)**	**1 (20.0)**	**2 (40.0)**
**Aminoglycosides**	**Amikacin**	**143**	**137 (95.8)**	**4 (2.8)**	**2 (1.4)**	**5**	**5 (100.0)**	**0 (0)**	**0 (0)**
	**Gentamicin**	**143**	**74 (51.7)**	**1 (0.7)**	**68 (47.6)**	**5**	**2 (40.0)**	**0 (0)**	**3 (60.0)**
**Carbapenems**	**Imipenem**	**142**	**137 (96.5)**	**2 (1.4)**	**3 (2.1)**	**5**	**5 (100.0)**	**0 (0)**	**0 (0)**
	**Meropenem**	**143**	**139 (97.2)**	**2 (1.4)**	**2 (1.4)**	**5**	**5 (100.0)**	**0 (0)**	**0 (0)**
**Third-generation cephalosporins**	**Ceftazidime**	**142**	**55 (38.7)**	**27 (19.0)**	**60 (42.3)**	**5**	**5 (100.0)**	**0 (0)**	**0 (0)**
	**Ceftriaxone**	**143**	**35 (24.5)**	**0 (0)**	**108 (75.5)**	**5**	**3 (60.0)**	**0 (0)**	**2 (40.0)**
**Penicillins**	**Amoxicillin + clavulanic acid**	**141**	**69 (48.9)**	**15 (10.6)**	**57 (40.4)**	**5**	**4 (80.0)**	**1 (20.0)**	**0 (0)**
	**Ampicillin**	**143**	**4 (2.8)**	**0 (0)**	**139 (97.2)**	**5**	**1 (20.0)**	**0 (0)**	**4 (80.0)**

^a^ Values are *n*(%). Adults are those aged ≥ 18 years. Paediatric cases are those aged < 18 years.

#### Staphylococcus aureus

**Fig. 2:**
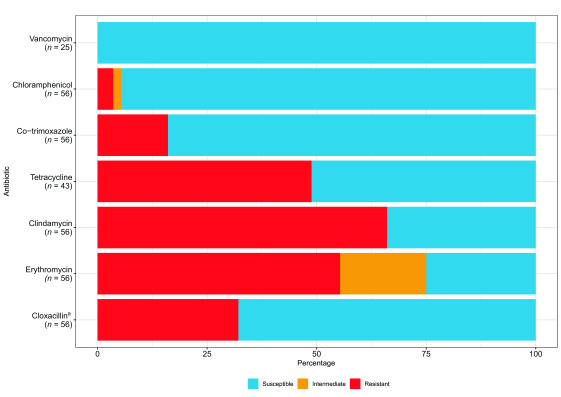
Antimicrobial resistance in Staphylococcus aureus blood isolates, Battambang, Cambodia, 2020–2022

After deduplication, there were 56 *S. aureus* blood culture isolates with AST data (**Fig. 2**). Resistance to methicillin was detected in around one third of isolates (18/56, 32%; 95% CI: 20–46%), but there was no evidence of resistance to vancomycin in the subset of isolates tested (0/25, 0%; 95% CI: 0–14%). AST results stratified by patient’s age are summarized in **Table 3**. There was no trend in methicillin resistance over time (44% [7/16] in 2020, 17% [3/18] in 2021, and 36% [8/22] in 2022; χ^2^ for trend *P* = 0.75) (data not shown).

[**insert** Figure 2]

**Table 3:** Antimicrobial resistance in *Staphylococcus aureus* blood isolates, stratified by patient’s age group, Cambodia, 2020–2022

Antimicrobial class	Antibiotic	Adult^a^				Paediatric^a^			
		Total	Susceptible	Intermediate	Resistant	Total	Susceptible	Intermediate	Resistant
**Glycopeptide**	**Vancomycin**	**23**	**23 (100.0)**	**0 (0)**	**0 (0)**	**2**	**2 (100.0)**	**0 (0)**	**0 (0)**
**Amphenicols**	**Chloramphenicol**	**44**	**42 (95.5)**	**1 (2.3)**	**1 (2.3)**	**12**	**11 (91.7)**	**0 (0)**	**1 (8.3)**
**Folate antagonists**	**Co-trimoxazole**	**44**	**36 (81.8)**	**0 (0)**	**8 (18.2)**	**12**	**11 (91.7)**	**0 (0)**	**1 (8.3)**
**Tetracyclines**	**Tetracycline**	**33**	**16 (48.5)**	**0 (0)**	**17 (51.5)**	**10**	**6 (60.0)**	**0 (0)**	**4 (40.0)**
**Lincosamides**	**Clindamycin**	**44**	**13 (29.5)**	**0 (0)**	**31 (70.5)**	**12**	**6 (50.0)**	**0 (0)**	**6 (50.0)**
**Macrolides**	**Erythromycin**	**44**	**10 (22.7)**	**9 (20.5)**	**25 (56.8)**	**12**	**4 (33.3)**	**2 (16.7)**	**6 (50.0)**
**Penicillins**	**Cloxacillin^b^**	**44**	**26 (59.1)**	**0 (0)**	**18 (40.9)**	**12**	**12 (100.0)**	**0 (0)**	**0 (0)**

^a^ Values are *n*(%). Adults are those aged ≥ 18 years. Paediatric cases are those aged < 18 years.

^b^ Cefoxitin was used as a surrogate disk to report methicillin resistance, with cloxacillin as the β-lactam reported to clinicians.

## Discussion

Analysis of blood culture data from BPRH for 2020–2022 revealed that the most frequently isolated pathogens associated with bloodstream infections were *E. coli* and *S. aureus*; resistance to key first-line antibiotics was common in both species. More specifically, our study found that 74% of *E. coli* isolates were resistant to ceftriaxone and that 32% of *S. aureus* isolates were resistant to methicillin, proportions that are considerably higher than the 48% and 22%, respectively, reported by a nongovernmental hospital-based study conducted in Phnom Penh in 2007–2010. (*8*) However, resistance rates were similar to those reported to GLASS in 2022, when data were pooled from Cambodia’s eight sentinel surveillance sites (including this hospital). According to the pooled data, 74% (95% CI: 69–79%) of *E. coli* isolates were resistant to third-generation cephalosporins and 68% (95% CI: 62–100%) of *S. aureus* isolates were methicillin-resistant.

The AMR rates at BPRH for 2020–2022 were higher than those reported to GLASS by adjacent countries. For 2022, Thailand reported rates of 34% (95% CI: 31–36%) for resistance to third-generation cephalosporins in *E. coli* and 8% (95% CI: 6–9%) for methicillin resistance in *S. aureus*, with Lao People's Democratic Republic reporting rates of 53% (95% CI: 46–59%) and 56% (95% CI: 39–72%), respectively. Unfortunately, Viet Nam did not submit AMR data for 2022 to GLASS. (*9*)

With the current data, it is difficult to make meaningful comparisons of AMR rates between countries in the Region. Inherent biases in surveillance data, which can arise from differences in patient populations and selective utilization of clinical diagnostic microbiology, (*10*) are likely to generate either under or overestimates of the true burden of AMR prevalence. In many locations, including Cambodia, AMR surveillance is in the early stages of implementation, and prevalence estimates are prone to these types of biases. Nevertheless, several factors may be more specific to Cambodia and may have a bearing on its rates of AMR. For example, in many parts of the country, it is common for patients to seek treatment from private health-care providers or use antibiotic self-treatment before being admitted to a government hospital. This practice may contribute to the selection of AMR in bacteria and thus overestimate the true prevalence of AMR in blood cultures, by inhibiting the growth of susceptible organisms. (*11*) Such practices may also be indicative of the wider issue of the inappropriate and excessive use of antibiotics. Om et al., for example, found that the drivers of AMR in Cambodia encompass the improper use of antibiotics in humans, (*12*) marked by excessive reliance on broad-spectrum antibiotics such as ceftriaxone. (*13*)

There are several limitations to this current study. First, the data cover only a 3-year period and involve a relatively small number of isolates, limiting their representativeness. In addition, the data set is a blend of community-acquired and hospital-acquired infections. Furthermore, the absence of clinical data and the lack of comprehensive information regarding the patient population present challenges in determining the impact of AMR.

However, the study also has several strengths. The blood culture positivity rate was 9%, suggesting that clinician uptake of diagnostic microbiology services was reasonable, and this reflects a positive attitude towards identifying bacterial infections in patients presenting to BPRH. However, confirming adequate coverage would require an audit of the clinical records of patients with relevant clinical syndromes to determine blood culture collection metrics. Other strengths include good laboratory practice: the hospital laboratory had an established quality management system in place and followed Clinical and Laboratory Standards Institute guidance for AST, including annually updating breakpoints.

Based on the findings, recommendations for further work can be suggested. The national AMR surveillance system should be strengthened to include patient-level data to improve understanding of the impact of resistance on clinical outcomes and to guide targeted interventions. Given the widespread misuse of antibiotics, it will be important to begin to monitor antimicrobial use and its appropriateness, at least at national sentinel sites for AMR surveillance. Surveillance data should be collated regularly and used to inform the development of or changes to treatment guidelines and to optimize empirical therapy. Given the issues around inappropriate antibiotic use in Cambodia, surveillance data should be used to raise public awareness about the seriousness of AMR and to promote the responsible use of antibiotics.

In conclusion, high rates of AMR were demonstrated in *E. coli* and *S. aureus* isolates from patients with bloodstream infections from a Cambodian provincial referral hospital. Further work is required to understand the clinical impacts of this resistance and to identify potential mitigation strategies.
